# A customised combination of environmental enrichment reduces aggression in CD-1 male mice

**DOI:** 10.1177/00236772251333986

**Published:** 2025-07-21

**Authors:** Amy Veness, Christophe Galichet, Sian Murphy, Tina O’Mahony, Yoh Isogai, Eleni M Amaniti

**Affiliations:** 1 Neurobiological Research Facility, Sainsbury Wellcome Centre, University College London, UK; 2 University Biomedical Services, 2152 University of Cambridge, UK; 3 Sainsbury Wellcome Centre, University College London, UK; 4 Allen Institute for Neural Dynamics, Seattle, USA

**Keywords:** Genetic background, animal welfare, mice, aggression, behaviour

## Abstract

Murine aggression has profound implications on animal welfare and husbandry. This report examines how three distinct combinations of environmental enrichment – wheel, igloo and tunnel; wheel, igloo, and tunnel with nesting; and tunnel with nesting – affect aggressive behaviour in CD-1 male mice. We found that combining wheel/igloo/tunnel enrichment with nesting or replacing the wheel/igloo with two tunnels while maintaining the nesting enrichment reduced aggression. These findings not only suggest how enrichment can improve the welfare of aggressive male mice but also emphasise the need for further research to determine the optimal combination of enrichment.

## Une combinaison personnalisée d’enrichissement environnemental réduit l’agressivité chez les souris mâles CD-1 Résumé

L’agression murine a des implications profondes sur le bien-être animal et l’élevage. Ce rapport examine comment trois combinaisons distinctes d’enrichissement environnemental – roue, igloo et tunnel; roue, igloo et tunnel avec nidification; et tunnel avec nidification – affectent le comportement agressif des souris mâles CD-1. Nous avons constaté que la combinaison de l’enrichissement roue/igloo/tunnel avec la nidification ou le remplacement de la roue/igloo par deux tunnels tout en maintenant l’enrichissement nidification réduisait l’agression. Ces résultats suggèrent non seulement comment l’enrichissement peut améliorer le bien-être des souris mâles agressives, mais soulignent également la nécessité de poursuivre les recherches pour déterminer la combinaison optimale d’enrichissement.

## Eine gezielte Kombination der Umweltanreicherung reduziert Aggression bei männlichen CD-1-Mäusen Abstract

Aggression bei Mäusen hat weitreichende Implikationen für Wohlergehen und Haltung der Tiere. In diesem Bericht wird untersucht, wie drei verschiedene Kombinationen von Umweltanreicherung – Rad/Iglu/Tunnel, Rad/Iglu/Tunnel mit Nistplatz sowie Tunnel mit Nistplatz – aggressives Verhalten von männlichen CD-1-Mäusen beeinflussen. Wir fanden heraus, dass die Kombination von Rad/Iglu/Tunnel mit Nistmöglichkeiten oder das Ersetzen von Rad/Iglu durch zwei Tunnel unter Beibehaltung der Nistmöglichkeiten Aggression reduzierte. Diese Ergebnisse lassen nicht nur darauf schließen, wie eine Anreicherung das Wohlergehen aggressiver männlicher Mäuse verbessern kann, sondern unterstreichen auch die Notwendigkeit weiterer Untersuchungen, um die optimale Kombination der Anreicherung zu erforschen.

## Una combinación personalizada de enriquecimiento ambiental reduce la agresividad en ratones machos CD-1 Resumen

La agresividad murina tiene profundas implicaciones en el bienestar y la cría de animales. Este estudio examina cómo tres combinaciones distintas de enriquecimiento ambiental (rueda, iglú y túnel; rueda, iglú y túnel con material para anidar; y túnel con material para anidar) afectan al comportamiento agresivo en ratones macho CD-1. Descubrimos que combinar el enriquecimiento con rueda/iglú/túnel con material para anidar o reemplazar la rueda/iglú por dos túneles manteniendo el enriquecimiento con material para anidar redujo la agresión. Estos resultados no solo sugieren cómo el enriquecimiento puede mejorar el bienestar de los ratones macho agresivos, sino que también ponen de manifiesto la necesidad de seguir investigando para determinar la combinación óptima de enriquecimiento.

## Introduction

Intermale aggression between cage-mates is a natural behaviour in mice essential for establishing hierarchy.^1^ However, aggression can cause mild signs, such as ruffled fur or minor wounds, to severe injuries requiring euthanasia. Standard husbandry practices, including removing the aggressor or single-housing all cage-mates, can mitigate harm but also raise welfare concerns owing to social isolation and increased stress.^2^

Aggression is influenced by multiple factors,^1,3–9^ including environmental enrichment^10,11^ and genetic background,^1^ yet the specific impact of enrichment remains unclear. Given the well-documented high aggression levels in CD-1 males^1^ which were housed in our facility, we report on the effects of three different enrichment conditions on their behaviour, that is, wheel/igloo/tunnel (W + TbE), wheel/igloo/tunnel plus nesting (W + T + NbE) and tunnel plus nesting (T + NbE). Less aggression was observed under W + T + NbE and T + NbE conditions. These results suggest that incorporating nesting materials plays a crucial role in mitigating conflict. Our findings highlight the importance of refining enrichment strategies to improve animal welfare aiming to preserve social housing.

## Animals, materials and methods

### Animals

CD-1/Crl (referred to as CD-1) and C57BL/6J male mice were imported from Charles River (UK) and kept in our AAALAC-accredited, UK Home Office-compliant, barrier-protected, specific-pathogen-free animal facility. Animal management was performed using PyRAT software (Scionics).

### Cage environmental enrichment

Mice were housed in controlled conditions (20–24°C, 45–65% humidity, 12 h light/dark cycle with 1h twilight) with ad libitum food (2016 Teklad, Envigo, UK) and reverse osmosis water in ventilated cages (Green/Emerald line, Tecniplast, UK). Bedding (Safe Bedding Asp, Aston Pharma, UK) and enrichment (Datesand, UK) included clear handling tubes, Fast Trac (wheel/igloo), aspen bricks, nestlet, tunnel clips, cardboard tunnels, and sizzle nest. Mice were housed in one of three conditions: W + TbE (aspen brick, wheel/igloo, clear tunnel), W + T + NbE (aspen brick, wheel/igloo, cardboard tunnel, nestlet, 10 g sizzle nest) or T + NbE (aspen brick, two cardboard tunnels, tunnel clip, nestlet, 10 g sizzle nest). Animals were monitored (visually) daily, with the animals removed from the cage and inspected weekly. Since the mice were not removed from the cage daily, we cannot rule out the possibility that the weekly inspections captured more severe aggressive encounters. Per UK Code of Practice, group-housed mice had ≥330 cm^2^ floor space, 45–60 cm^2^ per 20–30 g mouse, with a maximum of five per cage.

### Data extraction and analysis

CD-1 male aggression was analysed over 10 months using imported three-week-old CD-1 males for experiments. Data were collected during three periods: 2020/2021 (June to March, W + TbE: 569 C57BL/6J, 321 CD-1 mice), 2022/2023 (June to March, W + T + NbE: 371 CD-1 mice) and 2023/2024 (June to March, T + NbE: 314 CD-1 mice). Given that data were collected at different timepoints, which might imply different experimental conditions, no statistical test was performed; instead, the data collected are presented as absolute numbers.

Fight wounds, typically found on the back or tail and often accompanied by bleeding, were documented in PyRAT. To avoid duplicate entries, wounds were recorded only upon their initial discovery, with the experimental unit being the individual animal. The average age of the mice upon the initial discovery was 10.9 ± 5.53, 12.14 ± 5.35 and 14.6 ± 3.55 weeks for W + TbE, W + T + NbE and T + NbE, respectively. Mice were housed in groups of two to five, with average group sizes of 3.06 ± 0.99, 2.5 ± 1 and 4.33 ± 0.5 for the W + TbE, W + T + NbE and T + NbE conditions, respectively. The methodology did not account for the potential variation in incidence due to cage density effects. A scoring system for the severity of the wounds was not used. Known aggressors were isolated; otherwise, all males were singly housed. Superficial wounds were untreated, while deeper or clustered wounds received pain relief per veterinary advice. Males with exposed muscle were euthanized.

## Results

**Figure 1.**
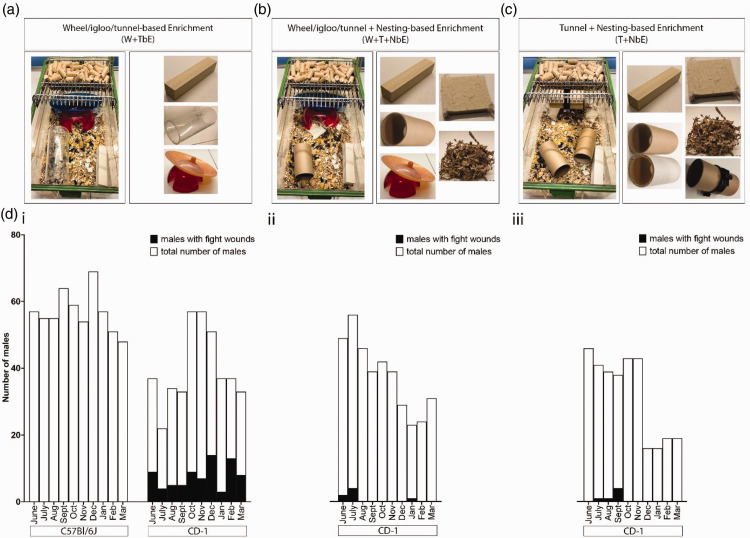
Environmental enrichment and male aggression. (a) W + TbE: wheel/igloo, plastic tunnel, aspen brick. (b) W + T + NbE: wheel/igloo, cardboard tunnel, aspen brick, 10g sizzle nest, nestlet. (c) T + NbE: two cardboard tunnels, tunnel clip, aspen brick, 10g sizzle nest, nestlet and (d) Fight wound data: total males (white) versus males with fight wounds (black) over 10 months. (di) C57BL/6J and CD-1 in W + TbE; (dii) CD-1 in W + T + NbE; (diii) CD-1 in T + NbE.

Between two and five CD-1 males were housed in W + TbE cages (Figure 1(a)). Over 10 months, 77 of 321 CD-1 males in W + TbE cages showed aggression-related wounds (Figure 1(di)), consistent with prior studies.^1^ In contrast, signs of aggression were absent in 569 C57Bl/6J males also housed in W + TbE cages (Figure 1(di)). We modified W + TbE cages by adding nesting material and replacing the plastic tunnel with cardboard (W + T + NbE; Figure 1(b)) or by adding nesting material, removing the wheel/igloo linked to increased aggression in CD-1 males^8^ and increasing the numbers of cardboard tunnels (T + NbE; Figure 1(c)). Among 371 CD-1 males in W + T + NbE cages and 314 in T + NbE, only seven and six males displayed fight wounds, respectively. The number of males with fight wounds was lower when compared with males housed in W + TbE cages (Figure 1(di) to (diii)).

## Discussion and conclusion

**Figure 2.**
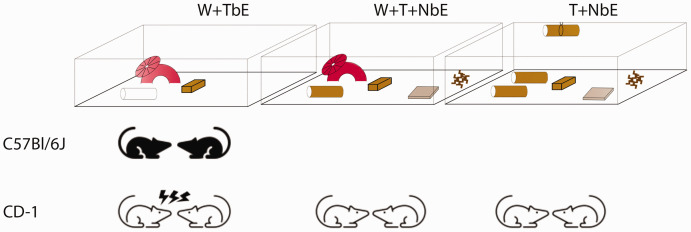
Schematic summary. Illustration of C57BL/6J and CD-1 male aggression in W + TbE (wheel/igloo, plastic tunnel, aspen brick), W + T + NbE (wheel/igloo, cardboard tunnel, aspen brick, 10g sizzle nest, nestlet) and T + NbE (two cardboard tunnels, tunnel clip, aspen brick, 10g sizzle nest, nestlet) housing conditions.

Aggression among co-housed male mice is a major concern in laboratory settings, resulting in stress, injuries and euthanasia. Current management strategies, such as removing aggressive mice or single-housing, are costly and increase staff workload. Therefore, optimising co-housing conditions is essential. CD-1 mice are particularly susceptible to aggression, and our report suggests that incorporating nesting materials, with or without a wheel or igloo, lowers this aggressive behaviour (Figure 2). Materials such as nestlets, sizzle nests and cardboard tunnels promote shredding, which may help redirect aggression. Future research could explore whether increasing access to these materials further encourages nesting behaviour and, hence, reduces aggression, as is the case for BALB/cAnNCRLBr.^12^

The enrichment conditions presented in this report were not tested concurrently. W + TbE condition was used during 2020–2021, coinciding with the reduced research activity during the COVID-19 pandemic. W + T + NbE and T + NbE were tested in subsequent years, potentially introducing confounding factors. Further studies with stress indicators and continuous cage monitoring to observe aggressive encounters directly are needed to determine whether transitioning to W + T + NbE or T + NbE effectively mitigates aggression, reducing the need for single-housing. This report has significant implications for managing aggression in co-housed male mice.

## Data Availability

To access data, interested parties can contact the corresponding author (e.amaniti@ucl.ac.uk). The data have not been deposited in repository.
